# Evaluating Young People's Ability to Sustain an Evidence-Based Social Accountability Approach to Improve Adolescent Sexual and Reproductive Health in Ntcheu, Malawi

**DOI:** 10.3389/frph.2021.645280

**Published:** 2021-07-01

**Authors:** Patience Mgoli Mwale, Thumbiko Msiska, Etobssie Wako, Kriss Chinkhota, Tapiwa Munthali, Mariela Rodriguez, Thembekile Shato, Anne Laterra, Anne Sebert Kuhlmann

**Affiliations:** ^1^CARE Malawi, Lilongwe, Malawi; ^2^CARE USA, Atlanta, GA, United States; ^3^Department of Behavioral Sciences & Health Education, College for Public Health and Social Justice, Saint Louis University, St. Louis, MO, United States

**Keywords:** social accountability, sustainability, evaluation, young people, Malawi, adolescent health, sexual and reproductive health

## Abstract

The Community Score Card^©^ (CSC), a social accountability approach, brings together community members, service providers, and local government officials to identify issues, prioritize, and plan actions to improve local health services. In addition, young people in Ntcheu, Malawi have been using the CSC approach to mobilize their communities to bring change across varying issues of importance to them. An earlier cluster randomized trial in Ntcheu showed the CSC effectively increased reproductive health behaviors, improved satisfaction with services, and enhanced the coverage and quality of services. Building upon this evidence of effectiveness, this study aims to evaluate if and how young people were able to sustain implementation of the CSC, and the improvements it brings, approximately 2.5 years after the randomized trial ended. As part of a larger evaluation of CSC sustainability in Ntcheu, we conducted 8 focus groups across 5 health catchment areas with 109 members of mixed-gender youth groups (58 females and 51 males, ages 14–29 years) who continued to engage with the CSC. Audio recordings were transcribed, translated into English, and coded in Dedoose using an *a priori* codebook augmented with emergent codes and a constant comparative approach. Although the 8 youth groups were still actively using the CSC, they had made some adaptations. While the CSC in Ntcheu initially focused on maternal health, young people adopted the approach for broader sexual and reproductive topics important to them such as child marriages and girls' education. To enable sustainability, young people trained each other in the CSC process; they also requested more formal facilitation training. Young people from Ntcheu recommended nationwide scale-up of the CSC. Young people organically adopted the CSC, which enabled them to highlight issues within their communities that were a priority to them. This diffusion among young people enabled them to elevate their voice and facilitate a process where they hold local government officials, village leaders, and services providers accountable for actions and the quality of healthcare services. Young people organized and sustained the CSC as a social accountability approach to improve adolescent sexual and reproductive health in their communities more than 2.5 years after the initial effectiveness trial ended.

## Introduction

Social accountability encapsulates both citizen engagement (the social) and governance (the accountability) (1). Social accountability approaches are mechanisms designed particularly to engage those systematically and historically excluded from power within communities, such as young people. These approaches strive to enable citizens a voice around issues related to quality, equity, and governance of local services by facilitating safe spaces in which to express that voice and negotiate actions to address concerns. While evidence supporting the effectiveness of social accountability overall has been building over the past decade (2–4), few social accountability approaches have focused explicitly on young people. As CARE was conducting a community randomized trial on social accountability in Malawi, an unexpected development emerged: without prompting or intention on behalf of the trial, groups of young people were adopting the social accountability approach and modifying it for their own use and objectives. This organic adoption of the approach offered an opportunity to explore not just how social accountability can engage youth meaningfully but how such engagement of young people could enhance the sustainability of these tools.

From a sustainability perspective, initiatives driven by young people that change power dynamics, governance structures, and expectations of the relationship between government and community have the potential to create fundamental, lasting structural change that improves health and development. Since young people are a dynamic, ever-changing segment of the population, it is important to understand their ability to pass along knowledge and ensure sustainability. Yet, few studies assess the sustainability of such interventions, and when they do they often do not address how the process supported sustainability (5). Understanding how and why interventions to improve sexual and reproductive health are sustainable is fundamental to our ability to create and replicate such initiatives across a variety of populations and contexts (6). Here, we look specifically at the role that young people can play in the sustainability of such a social accountability approach, what challenges they face in doing so, and what recommendations they have for the future.

While this special issue of the journal defines “young people” as individuals aged 10–24 years, which encompasses both “adolescents” (10–19 year olds) and “youth” (15–24 years) as defined by the United Nations, our work with young people encompasses those ranging from 14–29 years of age. We utilize the terms “youth” and “young people” interchangeably, both terms referencing individuals within this 14–29 age range.

Nearly two-thirds the population of Malawi, a country of nearly 16 million people, is 24 years old or younger (7). Women comprise 51% of the population, of which 45% are reproductive age (15–49) (8). HIV prevalence among the reproductive age population is 9.2% (9). Only 33.6% of adolescent females and 40.5% of adolescent males are enrolled in secondary school (10). The modern contraceptive prevalence rate for married women is 62%, but it is 48.3% for all women. The unmet need for modern contraceptives is 17.1% (11). Ntcheu district, in the Central Region of the country with a population of approximately 480,000, is a rural area; 85% of Malawi's population live in rural areas (8). Importantly, as part of its efforts to improve health and development, the Government of Malawi has prioritized engagement of young people in social policy-making generally, and sexual and reproductive health specifically, over the past couple of decades (12).

CARE Malawi, a non-governmental organization based in Lilongwe, Malawi since 1998 that works as part of the confederation model led by CARE International, originally developed the Community Score Card^©^ (CSC) in 2002 as a collaborative approach to social accountability (13). Initially designed for use in the health sector, it has since been used in the agricultural and education sectors, as well (14).

**Figure 1:**
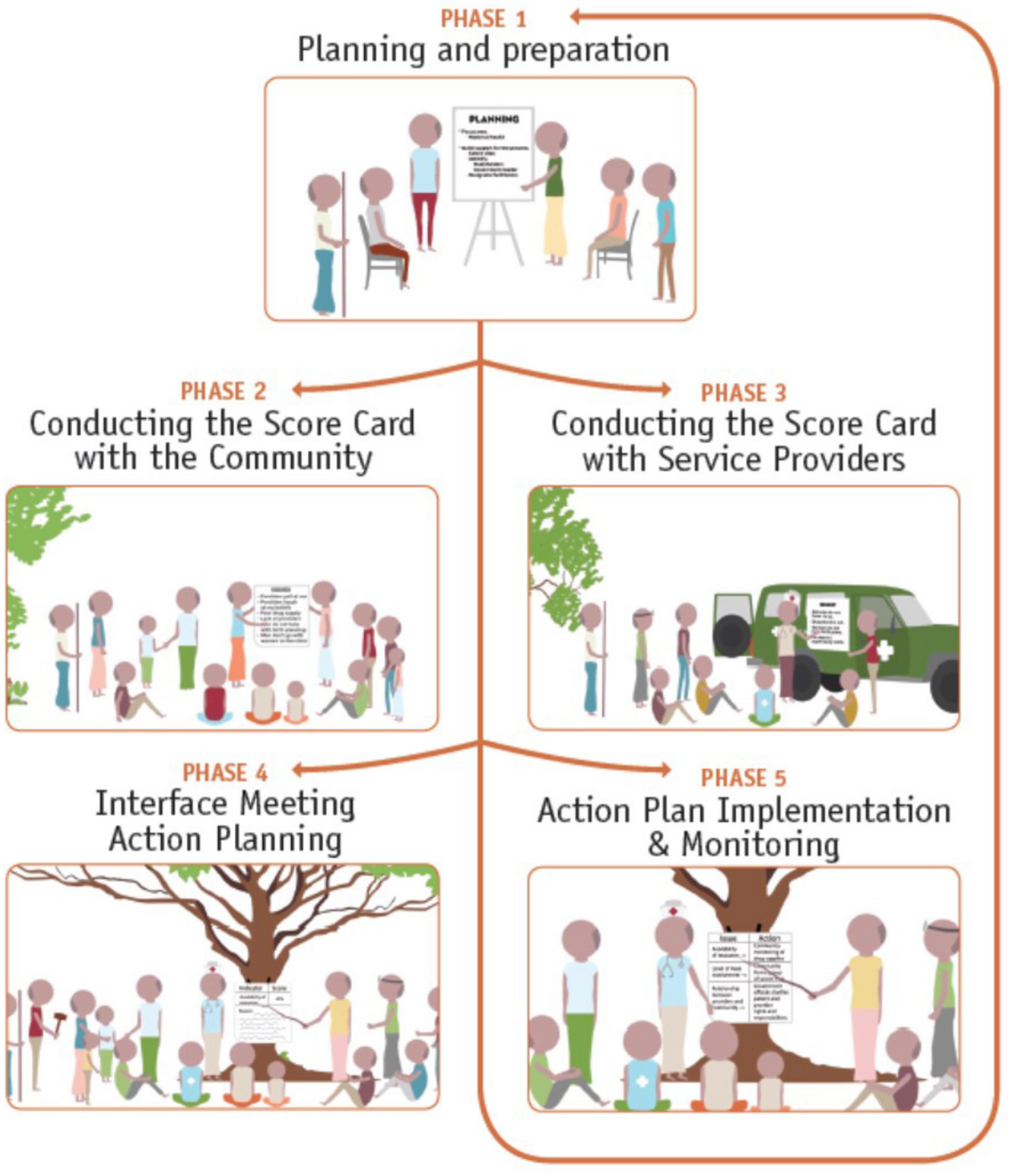
The Community Score Card^©^ (CSC) process.

The CSC brings together community members including young people, service providers, and local government officials for a facilitated process of issue identification, scoring and prioritization, interface and action step planning in a cycle that repeats every 6 months (see Figure 1). It gathers input from both service users and service providers to improve communication between communities, providers, and local government (13). The approach is inherently focused on the community, and the issues that are discussed and addressed during the process are community driven. The approach, which consists of five phases, has been described in detail elsewhere (15). Of the 5 phases, phases 2 and 3 focus on issue generation at the community level, led by community members themselves or service providers in the community (see Figure 1). Within phase 2, issues are raised by different members in the community—men, women, young people—these are issues that directly affect them and their utilization of services. Simultaneously, within phase 3, service providers in the community also discuss the issues that affect them—and their delivery of services. Throughout these phases and then through phases 4 and 5, the groups themselves (community members and service providers) prioritize the issues most important to them and come together during interface meetings to develop a joint action plan based on issues they have prioritized. Throughout the process, the community is the unit of analysis, and neither the authors nor CARE prioritized the issues that were generated or discussed throughout the process. Noteworthy about CARE Malawi's approach to social accountability is its emphasis on including both community members—particularly those historically excluded from consultative, decision-making or political processes such as young people—and frontline service providers as equal, collaborative stakeholders in the process and partners in demanding responsive action from higher-level decision makers and budget-holders.

During the Maternal Health Alliance Project (MHAP) from 2011–2015, CARE Malawi, together with the Government of Malawi's Ministry of Health, provided training on facilitation of the CSC process to community health action groups (CHAGs) across the 10 health facility catchment areas. Although MHAP did not purposively target young people to be trained in the facilitation of the CSC process, several young people happened to participate in some of these trainings as CHAG members. During this initial work with the CSC among CHAGs, these young people identified that they needed safe spaces to talk about their specific and unique health issues and needs. In response to this voiced need, CARE Malawi worked with the district government to establish youth clubs in 5 health center catchment areas, half of the intervention sites. These youth clubs were envisioned as a safe space for youth to come together, separate from adult members of the CHAGs or elsewhere. Once these clubs were established, however, many of the young people who had been trained through the CHAGs began introducing the CSC process to others in these youth clubs. While these youth clubs did receive support from CARE Malawi during the MHAP intervention period once they were formed, they were not part of the initial intervention plan and did not receive formal training as a separate stakeholder group in the CSC process. During the MHAP implementation, participation in the youth groups tended to range from about 10–15 members each. All groups were mixed-gender and mixed-age. Participants came from a wide variety of sources across the community, including existing youth clubs, religious organizations, community-based organizations, and youth development representatives.

A cluster randomized trial in Ntcheu from 2012–2014 showed positive effects of the CSC on community empowerment, service utilization, satisfaction with services, and improved service delivery and coverage among frontline health workers (15–17). While young people were part of the overall impact evaluation, there was not a separate impact evaluation just among young people. Building upon this earlier evidence of overall effectiveness—the *research*, this study aims to evaluate if and how young people sustain implementation of the CSC, and the improvements it brings, approximately 2.5 years after the initial trial—the *practice*. Here, we focus explicitly on the role of young people in the sustainability of the CSC, seeking to understand what drives the continuation of the CSC by groups of young people in Ntcheu district, Malawi approximately 2.5 years later.

## Materials and Methods

We designed an exploratory, cross-sectional study using qualitative methods to assess sustainability of various partner-led approaches (i.e., local government, young people, and CHAGs) of CSC implementation ~2.5 years after the end of the formal MHAP project. Here, we focus on that implementation and sustainability among young people. Focus group discussions allowed an in-depth exploration and better understanding of the uptake, implementation process, and outcomes of the CSC as experienced by young people in Ntcheu.

In mid-2018, around two and a half years after the end of MHAP and of formal support by CARE to facilitate the CSC process, we used purposive sampling to identify groups of young people across health center catchment areas who had been involved in the implementation of the CSC during the initial study. We conducted 8 focus groups with young people across 5 health center catchment areas with a total of 109 participants (*n* = 58 females and *n* = 51 males) in groups ranging in size from 8–18 participants each. Because the focus groups mirrored the composition of the youth groups that arose from the CSC process, all focus groups were mixed-gender with participants in the 14–29 age range. There was no difference in age and gender mix between groups that remained more active vs. less active. While we did not track educational status of individual participants, the median number of years of education for females in Ntcheu is 3.6 years; for males it is 4.1 years (8). Female literacy in Ntcheu is estimated at 74.8%, with estimates for male literacy around 88.9% (8).

Data collection was conducted by experienced researchers—fluent in both Chichewa (the local language) and English—from a Malawian research consulting firm. Prior to the start of data collection, facilitators and note-takers participated in a week-long training on both the CSC process and qualitative methods. Focus group discussions were held in the local primary health care centers and lasted between 60–90 min. Researchers obtained verbal informed consent from the participants prior to the start of the focus groups. Malawi's National Commission for Science and Technology reviewed and approved this study.

Focus groups were audio-recorded and transcribed in Chichewa and then translated into English by the data collectors in preparation for analysis. The authors then assumed responsibility for all coding and data analysis. An initial codebook with a small set of *a priori* codes was developed guided by the evaluation research questions. The codebook was then augmented and refined with emergent codes during the analysis process. Constant comparative analysis (18) was used to identify major themes arising through the analysis process, and supporting quotes were selected to help illustrate those themes. Constantly comparing data, as it is analyzed, to emerging themes or categories allows for the further refinement and definition of those themes throughout the analysis process. Per Boeije's (19) approach to constant comparative analysis, we first analyzed within the individual focus groups and then compared across cases of the same group (in this case, the youth groups), before finally looking across groups (i.e., other groups in the evaluation). Here, we focus on the analyses within the youth groups. Coding was done in Dedoose software. TS and ASK led the coding and checked for consistency across coders and cases. Analysis summaries by theme were drafted and shared among the research team members, discussed, discrepancies resolved, and then themes finalized. The final analytic themes were presented during a dissemination workshop in Lilongwe, Malawi in December 2019 that included young people who had participated in the youth focus groups. These final analytic themes are what are presented here.

## Results

Young people reported a mix of continued activity level around the CSC two and a half years after the end of the formal MHAP intervention period. Most groups of young people were still actively using the CSC process, although some with adaptations. Many groups liked how the CSC process facilitates young peoples' involvement in their communities and in development projects. Several groups talked about the CSC process as “research,” how it helps identify problems and negotiate between different constituents, and how it helps “track progress.” One group acknowledged, however, “being lazy since CARE left,” that is, not continuing to meet as frequently as they had previously during the formal intervention period.

“*community scorecard is a research method that stands for 2 parties, between … service user and the service provider, and find a way to solve that problem, that's an animal called scorecard”* [Chigodi (location of FGD), participant #1].

### CSC Process

Young people participating in the focus groups generally seemed to understand the CSC process, even though they had mostly taught each other and had not received formal training as a unique stakeholder group. They could describe the CSC process more than 2 years after the end of MHAP, and they continued to follow it. Focus groups discussed how the CSC process is beneficial for identifying issues, enabling differing segments of the population to use their voice (e.g., women, youth), and bringing different constituents together (e.g., teachers, health providers, community members). The indicator scoring process was well-understood and generally perceived to work well. Participants reported that interface meetings have been more challenging to sustain, however, because invited authorities might not show up or might deflect responsibility. Some groups explicitly discussed continuing to facilitate interface meetings while a few cited difficulties getting other stakeholders to attend such meetings as reasons for not continuing with that phase of the process.

Young people highlighted three important aspects of the CSC process as helping facilitate sustainability: (1) providing a *safe space* for them to speak out and building *agency* that enables them to work with various offices and be heard as an important stakeholder group in the community and to identify problems without appearing to assign blame; (2) creating *transparency* through conducting the scoring and prioritization phases in an open, public forum; and (3) generating *accountability* in monitoring community affairs through the development of actions plans that are monitored and reviewed every cycle (approximately every 6 months). Young people perceived that the CSC process has allowed them to gain a more active role in their communities and, specifically, with local development projects. Furthermore, it has helped them gain respect within their communities and be recognized as an important, unique stakeholder group. The structure of the CSC process in which groups are broken out for initial brainstorming and issue generation has provided young people a safe space to generate and share ideas. It has enhanced their ability to speak freely and have their voices heard. In their eyes, the CSC has improved relationships among various constituent groups in the local community.

“…*another thing I can talk about is that we are free, knowing each other, being free so that we can speak in public. At first it was difficult when we had not started scorecard for a person to stand in public in the village and speak as youth in our ages. We thought those who would speak in public were only the elders. But through scorecard we have benefited as youths to speak if we have observed a problem at a particular time in the village or in a family or wherever we have observed, we are able to stand and speak”* [Chigodi, participant #2]

“*…the scorecard whose benefits include transparency. There is participation of all when making decisions; while initially we were just told what to do. Scorecard helped to ensure that people are able to contribute ideas before a decision is made. There is sharing of knowledge and ideas so that everybody understands all community activities.”* [Manjanja-Kasinje, participant #1]

### Outcomes of the CSC for Young People

While maternal health was the primary focus of MHAP in Ntcheu, true to the nature and intent of the CSC, young people came to apply the CSC process to topics of importance to them, especially issues of sexual and reproductive health but also child marriages, student retention, and natural resources environment protection. Focus group participants mentioned a range of achievements that they credited to the CSC process, such as reducing early marriages and getting girls to return to school, better relationships between the health providers and community as well as teachers and community, fewer women delivering on the way to the hospital and more facility deliveries, and reduced deforestation and increased tree plantings. Specific focus groups mentioned achievements like getting the lab tech at the hospital to do a better job with patient confidentiality, helping elderly community members with house and farm work, and increasing contraceptive use and decreasing unintended pregnancies among youth. Many comments were about making people aware of the issues of importance to young people and getting village leaders and chiefs involved in promoting positive changes. Finally, young people felt the CSC has helped them uncover corruption and address environmental concerns in their communities such as deforestation. For example, one group explained how they uncovered corruption in the form of bags of porridge for students being pilfered off by school committee members and then were able, through the CSC process, to bring about change to end it.

“*…our group what we do, is to encourage the youth to take part in different positions/responsibilities as well as some of the things that we do pertaining to HIV and AIDS, just as said by this person that we also do environmental matters.”* [Champiti, participant #1]

“*As youth, it is not like we just tackle issues to do with the youth only, we also take care of the elderly like our parents and help them live healthy lives.”* [Nsiyaludzu, participant #1]

“*…here to help fellow youths how they can prevent contracting sexually transmitted infections… we ensure we take part to fight transmission of sexually transmitted infections and also in terms of development we ensure we reforest our area.”* [Chifwiri – Kasinje, participant #1]

One of the most important outcomes of the CSC was not, however, specific changes in health and development indicators, but instead, the perception of self-reliance that it creates among young people—the belief that they can and do have a role in the governance of their community, that they can affect change, and that they do not need to sit back and wait for others to address the issues that are of most importance to them as young people. They viewed this self-reliance as being built through the CSC's emphasis on skills, education, and advocacy. Young people viewed the CSC process as having helped them achieve greater independence along with a greater voice in community affairs. Overall, they felt the CSC helped empower and enlighten them.

“*…another thing is to enlighten youths so that they should be independent and self-reliant and also looking at our challenges and helping each other to address them in our area.”* [Yesaya-Kasinje, participant #1]

“*…the main objective is to empower young people to be self-reliant. We can also become self-reliant by skills and knowledge we normally share during our meetings as a youth club.”* [Manjanja-Kasinje, participant #2]

### Implementation Challenges Faced by Young People

The biggest challenge related to the CSC encountered by young people is convening and facilitating interface meetings with a variety of stakeholders. Some groups noted challenges around the involvement (or lack thereof) from officials and duty bearers—officers not showing up at the interface meeting, chiefs not promoting or supporting the meetings. Even when they were not able to implement the process with precise fidelity due to their inability to convene multi-stakeholder interface meetings, the youth did still complete the other phases of the process, focusing on actions that they could address themselves. These groups of young people are still using the CSC process to identify and prioritize issues of importance to them and to generate action plans to address these issues themselves as best they can, but they are not hosting and facilitating interface meetings regularly. In addition, not all groups followed the 6-month timeline for each cycle of the CSC process. The timeline for the different phases was a bit more fluid depending on what worked best for each group. Finally, a couple of groups reported challenges around transportation for members to attend meetings and resources such as refreshments, flipcharts, and markers to implement the process on their own.

“*Communication was already mentioned that our communication is a challenge as a result of transport. Because we come from far, even then going back we are supposed to walk to reach out homes, so, transport is a challenge. We longed if we had bicycles we would be meeting with ease.”* [Chigodi, participant #2]

“*Other challenges were to do with transport issues. Because some distances are really far, so we could not often manage to go because we did not have transport means.”* [Yesaya-Kasinje, participant #1]

While the principles and values underlying the CSC process were retained by the youth conducting the CSC, it was sometimes a challenge to implement the approach with complete fidelity to the original model. For example, without CARE to serve as a broker and facilitate, it made it difficult at times to get district-level stakeholders to attend meetings. While the implications of not having these district-level stakeholders could have meant that the CSC process was not as effective or was not able to mobilize desired changes in the community, it did show that the youth were still able to implement the process, focus on issues that were of utmost importance to them, and mobilize resources at the local-level, even if not at the district-level. Similarly, the youth groups did not always adhere to the 6-month timetable for cycles of the CSC process, but instead worked through the process on their own timeline. With these adaptations to the process, the youth were able to generate attention to issues that directly affected them and still find ways to bring about actions to solve some of the challenges they faced.

### Young People's Recommendations for the CSC

Overall, the CSC process has helped young people in Ntcheu take a more active role in the community and in development projects that affect them. It has helped them gain respect within their communities. Many groups emphasized how the CSC process has helped with the ability to speak freely, with transparency, and with accountability—all which has helped improve relationships between different community stakeholders. Young people recommended continued support for the CSC, especially in terms of material resources (e.g., markers, flipcharts, notebooks) to implement the process and refresher trainings on facilitating the process, at least during an initial transition period between fully-supported implementation and complete self-sustainability. All focus groups with young people recommended that CSC be implemented more widely throughout Malawi. They want nationwide scale-up of the CSC so others can benefit from and champion the CSC the way that they have.

The Community Score Card is a mechanism and a tool through which various stakeholders and various community members can voice concerns and issues; it enables them to jointly plan actions to solve them. The process of the CSC itself does not solve community problems. However, it is a mechanism that can fundamentally change how communities approach the challenges they face, and how they address those challenges. The principles and values that the CSC underscores are perhaps more meaningful that the actual process itself, and these principles are what leads communities, especially youth, to feel empowered to tackle the issues they face.

## Discussion

Meaningful engagement of youth is necessary to achieve high quality sexual and reproductive health for young people (20) which is why the Government of Malawi has prioritized such engagement in its policymaking, especially around adolescent sexual and reproductive health (12). Social accountability approaches, such as the CSC, present an opportunity to build this sort of meaningful engagement. Despite the call of multi-lateral organizations to utilize social accountability approaches to increase young people's engagement in health and development (21–24), only a few interventions have done so explicitly (25). Even fewer have assessed the sustainability of those approaches and sought to understand the process of sustainability, the mechanisms of how and why these approaches can be sustained.

The CSC process affords individuals the opportunity to voice concerns, challenges, and issues in spaces that were previously not available to them. The ability for marginalized groups to gather to generate issues and name service delivery challenges enabled different members of communities—not just community leaders—to participate. Once issues are generated, community members are also able to make decisions about which of the issues they think are most important. The gathering, collectivizing, and consciousness raising—first with peers, coupled with facilitated open dialogue with other stakeholders and constituents, enables individuals that may not usually be heard or given the opportunity to speak, to participate and elevate their voice. Individuals are not only able to make comments about the challenges and issues they face, but also participate in a process to score these issues in order to prioritize the ones they want to change and find solutions to. It is through the scoring process as well as action-planning phase of the process (see Figure 1) that many individuals feel they have decision-making power.

Here, building off research showing the effectiveness of the CSC (15–17), we have shown that in practice nearly two and a half years after the end of MHAP young people demonstrate robust knowledge and utilization of the CSC. This clearly illustrates knowledge retention of the CSC process over time, a key factor in sustainability. In addition, despite not having been formally trained in the process as a unique stakeholder group, young people are still engaged in the CSC process and continue to see results from their efforts. This also suggests knowledge about and belief in the CSC process is being passed from person to person, another key factor necessary for sustainability. Young people in Ntcheu are driving the CSC. It has been much more than just “engagement”; it has become a way to express agency and voice. The collective power, voice, and vision of young people in Ntcheu developed through the CSC demonstrate a promising opportunity for institutionalizing social accountability approaches among young people into communities and at all levels of government in Malawi and potentially elsewhere in sub-Saharan Africa. To our knowledge, there are no other studies of the sustainability of social accountability interventions this far after the end of the formal intervention period.

Young people leading the CSC process embodied the principles and values that the CSC manifests in communities—voice, empowerment, participation. In having the youth lead the CSC process among themselves, they were able to generate issues that affected them directly. They were also able to see tangible actions and changes take place such as staff attitudes shifting at health facilities which then increased the number of young people accessing health services and receiving youth-friendly services. They observed reduce forced, early, and child marriages and increased efforts to ensure girls could go back to school after marriage or childbirth. These were all changes that affected youth directly and were a result of the direct engagement young people had on issues that affected them.

This study highlights barriers and facilitators of sustainability—practical considerations that can guide future social accountability approaches among young people. While the young people struggled at times with convening and facilitating interface meetings with multiple stakeholders, it is the safe space and agency afforded by the process through which young people witness transparency and accountability happening that keeps them motivated to continue using the CSC process, even if they are doing so in a somewhat adapted form. Future practice-oriented research around social accountability among young people should test the ability of train-the-trainer models to engage emerging, new leaders effectively on a continual basis and to test a formal transition phase between full-supported implementation and complete self-sustainability by young people. In addition, those implementing social accountability approaches with an eye toward long-term sustainability should consider how best to prepare others for a time when marginalized or vulnerable groups within the community, such as young people, will be responsible for initiating and facilitating interface meetings that involve a range of stakeholders across the community. Planning for this in advance is critical as interface meetings are, in many ways, at the heart of the CSC approach. Without the opportunities created by these meetings for duty bearers and rights holders to come together, the CSC process risks becoming a one-sided, strategic planning and prioritization process, not an accountability one. This may require implementing organizations, like CARE, to step-back earlier in the CSC process and think critically about how they are facilitating access to these spaces for youth, and other historically excluded groups, in a way that builds their credibility and stakeholders responsiveness to their requests long before project end dates.

## Limitations

Several limitations of this study should be acknowledged while also recognizing its strengths. First, those young people interested in participating in focus groups about the CSC nearly two and a half years after the end of MHAP are, by definition, more engaged in the process than those who did not attend. Over 100 participants across 8 focus groups in 5 health center catchment areas did attend, however, suggesting broad continued support of and interest in the CSC process. Second, while this was a cross-sectional study capturing a single point in time, sustainability is dynamic. Momentum for engagement in the CSC process among young people in Ntcheu could either continue to build or wane in the future, particularly when faced with emerging challenges like the COVID-19 pandemic which has both made facilitating processes like the CSC more challenging yet even more critical. We know, however, that the CSC was being actively sustained by young people in Ntcheu nearly two and a half years after the end of MHAP. There are relatively few studies of sustainability, and those that there are often look at sustainability 6- or 12-months post-interventions (5). Here, we present evidence of sustainability at a much further time point post-intervention. The large number of young people still actively engaged in the process more than 2 years after the end of the formal intervention suggests the power of the CSC to instill lasting change and the value that young people continue to see in the approach.

## Conclusions

Young people in Ntcheu provide important insights into why and how the CSC process is sustainable and brings about change. They emphasize the *safe space* created by the process to allow young people to voice their opinions first amongst themselves and then as a unique, important stakeholder group to other constituents in the community, the *transparency* of the process afforded by the scoring and prioritization process and the rationale for the scoring done publicly, and the *accountability* provided by having a public, documented action plan that is monitored by the group and revisited every cycle (~every 6 months). These are concrete components of the process that other social accountability interventions among young people can replicate to increase sustainability. Furthermore, the CSC process builds a sense of self-reliance among young people. It is important to recognize, however, that through maturation the cohort of young people in the district is continually changing so sustainability requires having new leaders emerge and be trained in the CSC process by existing cohort members. Again, interventions can plan for this maturation factor by incorporating train-the-trainer type models and periodic refresher trainings during a transition period from fully-supported implementation to complete self-sustainability into their initial intervention plans. The hope and expectation is that these types of social accountability approaches with young people can help address the needs and gaps of sexual and reproductive health, that they can help bring long-term structural change related to engagement with and governance of services in order to create institutions that work better for and with the young people they aim to serve.

## Data Availability Statement

The raw data supporting the conclusions of this article will be made available by the authors, without undue reservation.

## Ethics Statement

This study involving human participants was reviewed and approved by Malawi's National Commission for Science and Technology. Verbal informed consent was obtained. Written informed consent for participation was not required for this study.

## Author Contributions

PM, TMs, KC, and TMu oversaw CSC implementation and data collection and contributed to protocol development and data analysis. EW, MR, TS, and AL contributed to protocol development and data analysis. ASK oversaw protocol development and data analysis. All authors contributed to manuscript development and reviewed and approved the final manuscript.

## Conflict of Interest

The authors declare that the research was conducted in the absence of any commercial or financial relationships that could be construed as a potential conflict of interest.
